# Thermohaline structure and circulation beneath the Langhovde Glacier ice shelf in East Antarctica

**DOI:** 10.1038/s41467-021-23534-w

**Published:** 2021-07-09

**Authors:** Masahiro Minowa, Shin Sugiyama, Masato Ito, Shiori Yamane, Shigeru Aoki

**Affiliations:** 1 Institute of Low Temperature Science, Hokkaido University, Sapporo, Japan; 2 Present Address: Japan Agency for Marine-Earth Science and Technology, Yokosuka, Japan

**Keywords:** Cryospheric science, Physical oceanography, Cryospheric science

## Abstract

Basal melting of ice shelves is considered to be the principal driver of recent ice mass loss in Antarctica. Nevertheless, in-situ oceanic data covering the extensive areas of a subshelf cavity are sparse. Here we show comprehensive structures of temperature, salinity and current measured in January 2018 through four boreholes drilled at a ~3-km-long ice shelf of Langhovde Glacier in East Antarctica. The measurements were performed in 302–12 m-thick ocean cavity beneath 234–412 m-thick ice shelf. The data indicate that Modified Warm Deep Water is transported into the grounding zone beneath a stratified buoyant plume. Water at the ice-ocean interface was warmer than the in-situ freezing point by 0.65–0.95°C, leading to a mean basal melt rate estimate of 1.42 m a^−1^. Our measurements indicate the existence of a density-driven water circulation in the cavity beneath the ice shelf of Langhovde Glacier, similar to that proposed for warm-ocean cavities of larger Antarctic ice shelves.

## Abstract (web summary)

Basal melting of ice shelves is the principal driver of recent ice mass loss in Antarctica. The study reports comprehensive structures of temperature, salinity and current under an ice shelf in East Antarctica obtained by borehole measurements.

## Introduction

The Antarctic Ice Sheet feeds ice into the ocean through fast-flowing outlet glaciers and ice streams^1^. The glaciers and ice streams form floating ice tongues and ice shelves, accounting for 74% of the coastal margins of the ice sheet^2^. Because approximately half of the ablation of the Antarctic Ice Sheet occurs due to basal melting of floating ice^3,4^, processes at the ice–ocean interface are very important for the ice sheet mass budget. Furthermore, thinning or loss of an ice shelf causes reduction in the buttressing force acting on inland ice. This accelerates ice discharge into the ocean, as observed in the Antarctic Peninsula region^5^. The most rapidly melting ice shelves are observed in the Bellingshausen–Amundsen seas sector of West Antarctica^6^. There, warm and dense circumpolar deep water (CDW) with a temperature of ~1°C (~3°C above the in situ freezing point) flows into subshelf cavities, supplying heat that enables rapid melting^7^. Observations and numerical modeling have indicated strong temporal and spatial variability in the basal-melt rates of ice shelves in the Bellingshausen and Amundsen seas^8,9^, where mass loss of the ice sheet has recently been increasing^10^. Recent observations at Totten Glacier demonstrated that rapid ice shelf base melting occurs in East Antarctica as well^11,12^. Heat for the basal melting in the Totten Glacier region is carried by modified CDW, which is slightly colder than CDW but still sufficiently warm for melting.

Circulation beneath these rapidly melting ice shelves is driven by an out-flowing buoyant plume generated by basal melting. This density-driven circulation allows for the advection of warmer, saltier, and denser water to the grounding line. The basal-melt rate $\dot{m}$ is commonly estimated from ocean temperature *T*, salinity *S*, and current speed *U* in the boundary layer beneath an ice shelf^13^ (see full equations in “Methods”)

1 $$\dot{m}=f(U,\ T-{T}_{\mathrm{f}},\ S),$$

where *T*_f_ is the freezing point of the seawater in the mixed layer beneath the ice shelf, but calculated using the pressure of the ice shelf base. Therefore, direct measurements of temperature, salinity, and water current underneath an ice shelf are crucial to estimate the melt rate based on physical processes at the ice–ocean boundary. In addition, in situ oceanic data from a subshelf cavity provides critical information for furthering our understanding of basal melting and water circulation. The equation for estimating melt rate assumes the transfer of ocean heat from a fully turbulent boundary layer to a hydraulically smooth ice shelf base^13^. However, turbulence in the boundary layer and the roughness of the ice-surface topography can only be investigated using direct measurements.

Subshelf density-driven circulation is categorized into three major modes, which are associated with the magnitude of ocean heat transport into a subshelf cavity^14^. In Mode 1, salty water at freezing points is generated by brine rejection during sea ice production and flows into the subshelf cavity. Since the freezing point drops as pressure increases, heat is available in the water to melt ice at the depth of the ice shelf base and a part of the upwelling cooled water might refreeze before it exits the cavity. In Mode 2, warm CDW flows onto the continental shelf and into the cavity. CDW causes rapid basal melting since it is warmer than the freezing point at the ice shelf base, typically by ~3°C. In Mode 3, Antarctic Surface Water (ASW) carries heat supplied by the atmosphere into the cavity, causing a significant amount of basal melting near the ice shelf front during the summer^15,16^. Although these modes are widely accepted in the community, in situ oceanic data with coverage of an entire cavity is required for validation of the proposed processes.

Studies have shown melting near the ice shelf grounding lines to be a key for the future evolution of the ice sheet^17^. However, understanding of mixing processes in the vicinity of grounding lines is limited due to the difficulty of obtaining measurements. Theoretical studies suggest that tidal currents erode stratification and induce vertical mixing near the grounding line^18^. On the other hand, recent observations found a lack of tidal mixing in the grounding zone of Ross Ice Shelf, where vertical mixing was instead driven by relatively inefficient diffusive convection^19^. To better understand the processes controlling basal melting, in situ observations of thermohaline structure, and circulation regimes will be required over extensive areas of a subshelf cavity.

In this study, we made direct measurements of ocean properties beneath the ice shelf of Langhovde Glacier, East Antarctica in January 2018, using boreholes drilled with a hot-water system through ice 234–412-m thick. Four boreholes were located along a glacier flowline from the ice shelf front to the grounding line. Water temperature, salinity, and currents were measured in the subshelf ocean cavity, which had thicknesses between 302 and 12 m. Our observations confirmed the advection of warm water into the cavity, and revealed the circulation regime under the ice shelf of Langhovde Glacier. Based on these data, we quantify the rate of basal melting and the stability of the water column, providing an overview of the ice–ocean interaction of the ice shelf of a small outlet glacier in East Antarctica.

## Results

### Study site

**Fig. 1:**
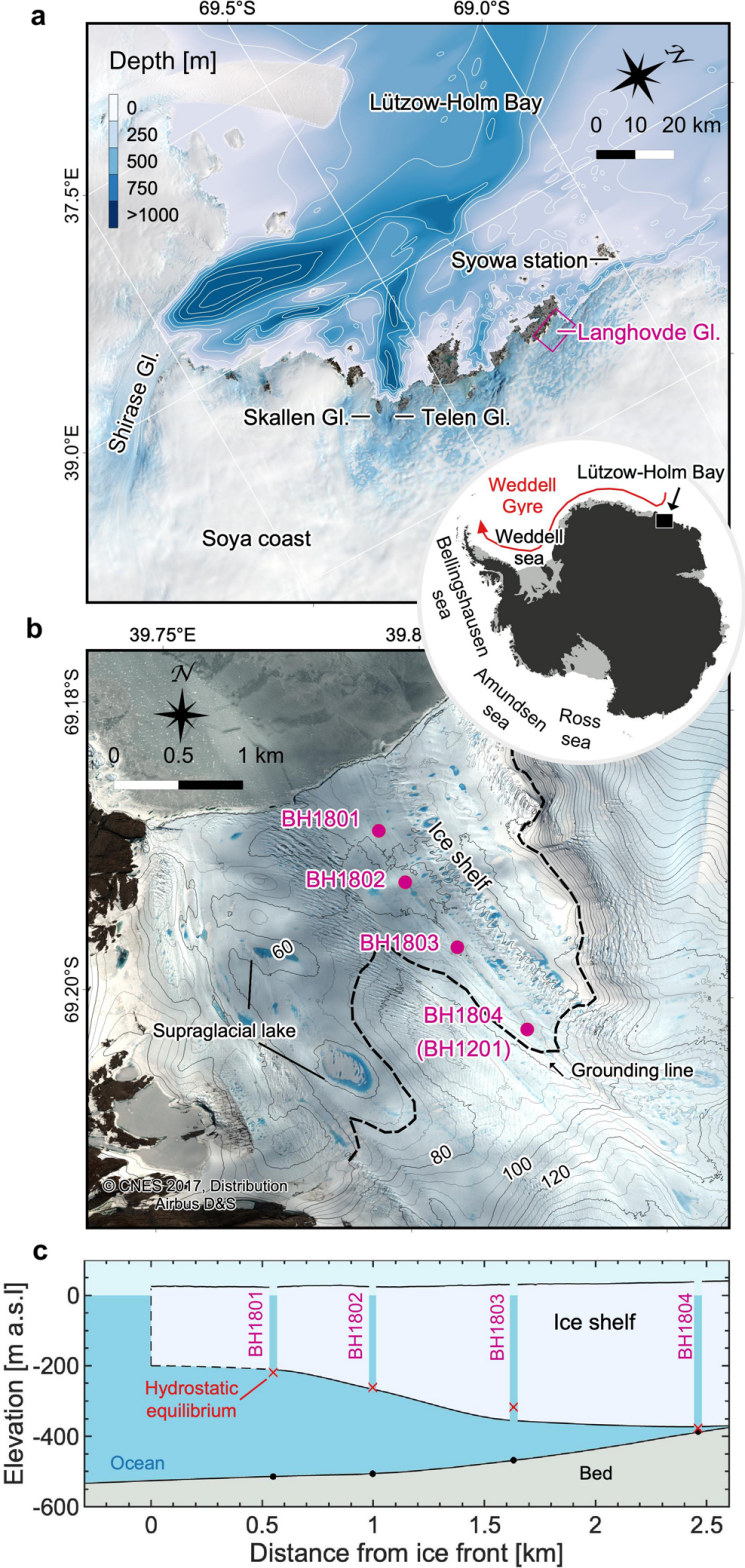
Study site. **a** Satellite image of Lützow-Holm Bay obtained by Landsat 8 on 11 November 2017. The contour lines indicate the ocean depth with intervals of 200 m. The location of Lützow-Holm Bay in Antarctica is indicated in the inset. Red arrow is a schematic representation of the Weddell Gyre after Fig. 7 in ref. ^25^. **b** Locations of the drilling sites along the ice shelf of Langhovde Glacier (purple dots). The image and surface elevation were acquired by Plèiades on 21 December 2017. The surface elevation contour lines are every 5 m. Black dashed curve indicates the grounding line estimated by the break in slope method^20^. **c** Vertical cross-section of Langhovde Glacier along the boreholes. The red crosses are the ice bottom elevation estimated from the surface elevation based on the hydrostatic equilibrium assumption. The surface elevation (black contour) was obtained from elevation model of Plèiades^47^. The lower boundary of the ice shelf and the ocean bed were drawn by interpolation of borehole measurements and bathymetry map in front of the ice shelf.

Langhovde Glacier is a 3-km wide outlet glacier draining into Lützow-Holm Bay in East Antarctica (Fig. 1). The lower 10 km of the glacier flows in a channel, which is bounded by bedrock to the west and by slowly moving ice to the east (Fig. 1). In this channelized region, ice near the ice shelf front flows at a rate of ~150 m a^−1^ (ref. ^20^), modulated by tides^21^. Surface topography indicates that the glacier has a several kilometer long ice shelf with a grounding line protruding toward the ocean in its central part^22^ (Fig. 1b). The grounding zone of the glacier was drilled in a previous field campaign, which confirmed a 10-m-thick ocean-water layer below 430-m-thick ice at 3.1 km from the ice shelf front^23^. The grounding line location was estimated to be several hundred meters upglacier from the drilling site.

The bathymetry of Lützow-Holm Bay was also surveyed during previous expeditions^24^. A ≥1-km deep ocean trough is incised into the continental shelf and continues to Shirase, Skallen, and Telen Glaciers, which are major outlet glaciers terminating in the bay (Fig. 1a). Another trough extends to Langhovde Glacier along the Soya Coast through a passage between the ice sheet and Ongul Islands, where Syowa Station, a Japanese research base, is located (Fig. 1a). The depth of the ocean in front of the glacier is ~600 m, but ocean bed topography below the Langhovde Glacier ice shelf is unknown.

Lützow-Holm Bay is located near the eastern boundary of the Weddell Gyre, where the Antarctic Circumpolar Current approaches the coast^25^ (Fig. 1). The clockwise circulation of the gyre and coastal current carry Warm Deep Water (WDW) along the continental slope, which supplies heat for the melting of the ice sheet’s margins^15,26^. WDW flows into the main trough of Lützow-Holm Bay^27^, resulting in significant basal melting of Shirase Glacier^28,29^. A mooring measurement under Fimbul Ice Shelf in Dronning Maud Land showed that the cavity was occasionally accessed by Modified Warm Deep Water (MWDW), which is a mixture of WDW and Winter Water (WW)^15^.

### Ice and bed geometries

Borehole drilling revealed ice and ocean bed geometries of the Langhovde Glacier ice shelf at the drill sites (Fig. 1c and Table 1). At the northernmost borehole BH1801 (0.5 km from the front), 234-m thick ice was underlain by a 302-m-deep seawater column. The ice thickness increased toward the grounding zone, i.e., 294 m at BH1802, 389 m at BH1803, and 412 m at the uppermost borehole BH1804 (2.4 km from the front). Bed elevation increases and the water layer thins toward the grounding zone (Fig. 1c). At BH1804, the bed elevation was 364 m below sea level and the subshelf water layer was 12-m thick.

**Table 1:** Summary of ice and bed geometries at the drilling sites.

Borehole		BH1801	BH1802	BH1803	BH1804
Drilling date		Jan-16, 2018	Jan-9, 2018	Dec-31, 2017	Jan-22–23, 2018
Ice thickness	m	234	294	389	412
Ice-surface elevation	m a.s.l	21.7	25.8	31.5	37.3
Bed elevation	m a.s.l	−514.7	−507.8	−468.0	−387.8
Ice thickness from hydrostatic equilibrium assumption	m	241	287	349	414

Ice thickness measured at boreholes BH1801, 02, and 04 agrees with those calculations based on the hydrostatic equilibrium assumption to within 3% (Fig. 1c and Table 1). Nevertheless, ice thickness at BH1803 was greater than the estimation by 40 m, showing that the buoyant force at the drill site exceeded the gravitational force by 10%. The ice was being depressed by stresses acting from ice surrounding the site.

**Fig. 2:**
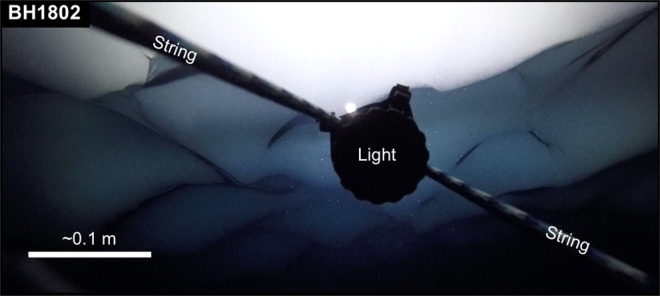
Ice-surface geometry. Lower surface of ice at BH1802 as observed by an upward-looking camera (see also Supplementary movie).

An upward-looking video camera was lowered into the borehole at BH1802 to inspect the lower surface of the ice shelf (Supplementary movie). The surface was covered with dimples with a diameter of 0.2–0.3 m and depth of 0.05–0.1 m (Fig. 2).

### Subshelf ocean properties

**Fig. 3:**
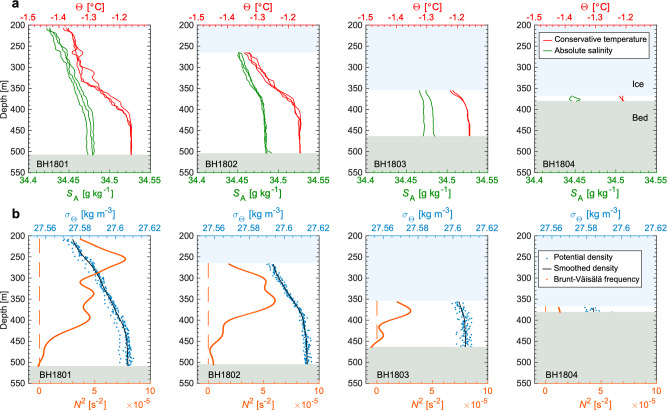
Subshelf ocean properties. **a** Conservative temperature, Θ, (red) and absolute salinity, *S*_A_, (green) profiles measured at the borehole sites. Water properties were measured twice or three times at each site around high and low tides (Supplementary Fig. 2). Light blue and gray shades indicate ice and seabed, respectively. **b** Potential density, *σ*_Θ_, (blue) and the square of Brunt-Väisälä frequency, *N*^2^, (orange) as means of all measurements at each site.

Oceanographic measurements were repeated 2–3 times at each borehole, so that conditions were observed during both high and low tides (Supplementary Fig. 2). Conservative temperature (Θ) in the cavity ranged between −1.4 and −1.15°C (Fig. 3a). Conservative temperature and absolute salinity (*S*_A_) increased from the ice to the seafloor (Fig. 3a). In the vicinity of the ice–ocean boundary, the water temperature was above the in situ freezing point by 0.65–0.95°C (Fig. 3a and Supplementary Fig. 4). Water properties were nearly homogeneous below 420 m. The average conservative temperature and absolute salinity within the layer below 420 m were −1.18°C and 34.48 g kg^−1^, respectively. Near the grounding line at BH1804, water was colder and fresher than that observed at the same depth in the other profiles by 0.05°C and 0.04 g kg^−1^ (Fig. 3a).

Brunt-Väisälä frequency (stability of water stratification, *N*) showed local maxima at 18–48 m below the ice base (Fig. 3b, Table 2, and Supplementary Fig. 3), which we assume to mark the lower boundary of the ice–ocean boundary layer. Salinity within the boundary layer was similar at the four sites, whereas temperature near the grounding line (BH1803–04) was more than 0.1°C higher than closer to the front (BH1801–02) (Table 2).

**Table 2:** Summary of in situ observations in the ice–ocean boundary layer: mean temperature (*T*), absolute salinity (*S*_A_), pressure (*P*), current (*U*) averaged within the ice–ocean boundary layer (*D*), and computed basal-melt rate ($\dot{m}$).

Borehole	Date	Start time	End time	*T*	*S*_A_	*P*	*U*	*D*	$\dot{m}$
		UTC	UTC	°C	g kg^−1^	MPa	mm s^−1^	m	m a^−1^
BH1801–H	Jan-17, 2018	15:16	15:23	−1.36	34.43	2.15	14.1	42	1.42
BH1802–H	Jan-10, 2018	10:15	10:21	−1.30	34.46	2.75	4.8	40	0.56
BH1803–H	Jan-01, 2018	17:21	17:27	−1.20	34.47	3.68	8.7	18	1.29
BH1804–H	Jan-24, 2018	07:00	07:02	−1.21	34.45	3.81	8.4	12	1.25
BH1801–L	Jan-17, 2018	10:13	10:21	−1.35	34.43	2.15	16.3	47	1.62
BH1802–L	Jan-10, 2018	15:24	15:31	−1.31	34.45	2.75	8.8	48	1.00
BH1803–L	Jan-06, 2018	07:15	07:21	−1.20	34.48	3.68	17.0	18	2.46
BH1804–L	Jan-23, 2018	12:15	12:18	−1.20	34.44	3.81	12.0	12	1.77
Mean									1.42

Measurements were taken at least twice at each drilling site (BH1801–BH1804) around high tide (denoted by H) and low tide (denoted by L). The listed water properties were used to calculate the basal-melt rates.

**Fig. 4:**
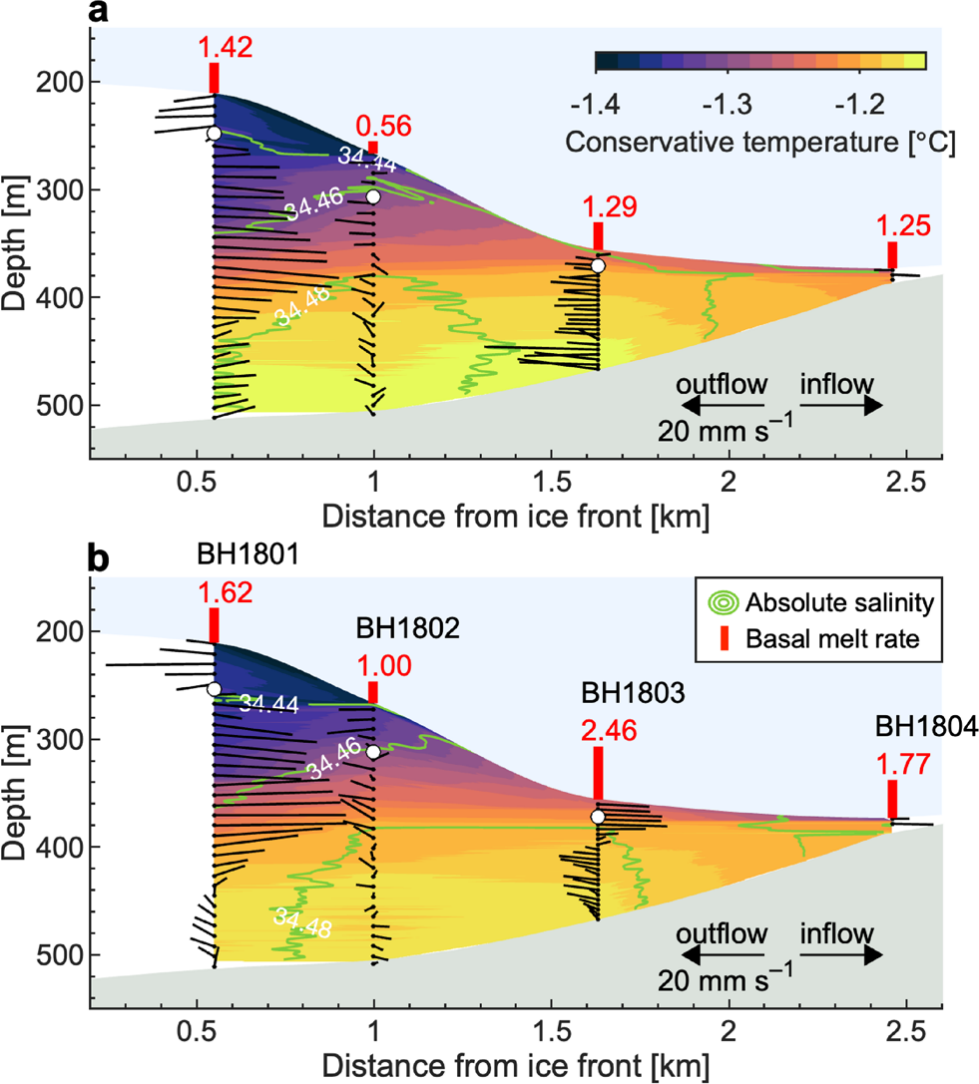
Overview of thermohaline structure and circulation. Water current, conservative temperature, and absolute salinity measured in the ice shelf cavity of Langhovde Glacier in January 2018. Measurements were performed twice at each borehole site in (**a**) high and (**b**) low tides. The length and orientation of black lines indicate current speed and direction. Cross-glacier components of the current speed are projected to the vertical: leftward-looking—flow to the open ocean, rightward-looking—flow to the grounding zone, upward-looking—flow to the northeast, and downward-looking—flow to the southwest of the cavity on the plane view (Fig. 1b). Background shows the vertical cross sections of conservative temperature (color scale) and absolute salinity (green contour line). Estimated basal met rates (m a^−1^) are indicted by red bars and numbers. Open circles indicate the lower boundary of the ice–ocean boundary layer.

At BH1801, water flowed toward the open ocean within ~50 m of the ice base, whereas a relatively strong current (40 mm s^−1^) toward the grounding line was observed at a depth of 260–420 m. The flow below 420 m was relatively weak (10–30 mm s^−1^) and changed its direction between high and low tides (Fig. 4). At BH1802, water in the boundary layer flowed downglacier at a rate of 20 mm s^−1^. Below the boundary layer, current deviated eastward at a rate of 10 mm s^−1^ (Fig. 4). At BH1803 and 04, the magnitude and direction of the water current was substantially different between the casts performed at high and low tides. For example, at BH1803, the entire water column flowed toward the glacier front during high tide (Fig. 4a), whereas flow in the upper layer switched toward the grounding line during low tide (Fig. 4b). Water current generally increased toward the front, except for the relatively weak flow observed at BH1802 (Table 2). The observed range of water current (10–40 mm s^−1^) corresponds to a travel time of 3–0.7 days between the ice shelf front and the grounding zone.

## Discussion

**Fig. 5:**
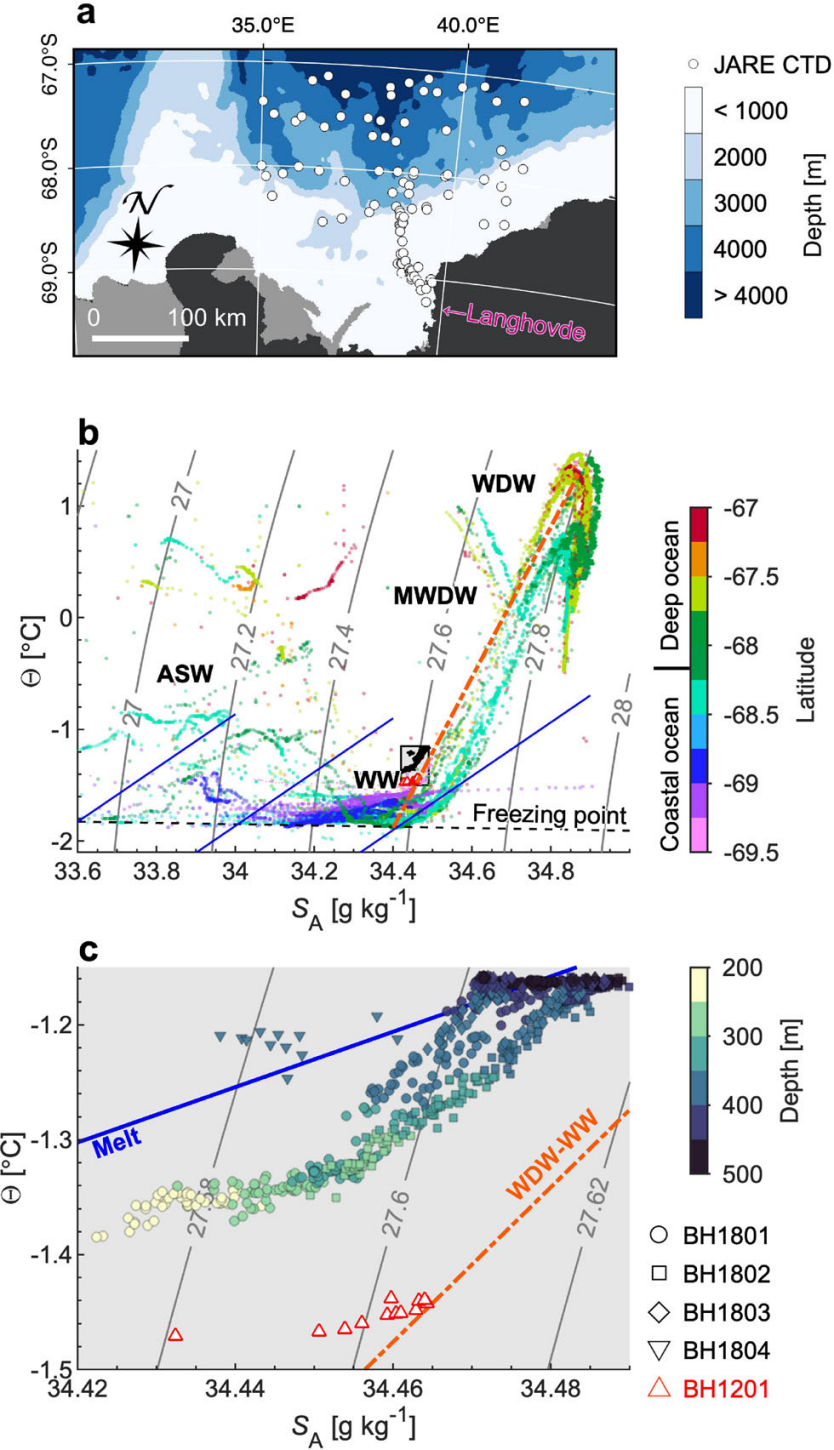
Ocean and subshelf water properties. **a** Locations of the CTD casts (white circles) previously performed by the Japanese Antarctica Research Expedition. The background bedrock topography is from the IBCSO bathymetry^48^. **b** Conservative temperature-absolute salinity diagram, showing water properties previously reported in the nearby ocean, and those obtained beneath the ice shelf of Langhovde Glacier in this study (black dot) and January 2012 at BH1804 (red triangle)^23^. Locations of the CTD casts in the ocean are indicated in Fig. 5a. Also indicated are the meltwater-mixing line^44,45^ (thick solid blue; gradient of 2.41°C/(g kg^−1^)), isopycnals (gray), freezing point (dashed black) referenced surface pressure, and the WDW-WW mixing line (dash-dotted orange). The box bounding the subshelf measurements indicates the region enlarged in (**c**). **c** Conservative temperature and absolute salinity obtained by subshelf measurements in this study. Marker color represents the depth of the measurement. See Supplementary Fig. 4 for more details of the water properties near the ice–ocean boundary. WDW: Warm Deep Water, MWDW: Modified Warm Deep Water, WW: Winter Water, and ASW: Antarctic Surface Water.

The water temperature in the ice–ocean boundary layer exceeded the in situ freezing point at all the boreholes (Fig. 3), indicating that the ice was subjected to basal melting from the grounding line to the front. Water properties within the ice–ocean boundary layer generally lie along the meltwater-mixing line except for a thin 2–3-m thick water layer immediately below the ice (Fig. 5c and Supplementary Fig. 4b). Basal-melt rates were estimated by solving the heat and salt conservation equation (Eqs. (3)–(6))^13^ using previously proposed parameters^30^ (Supplementary Table 1). The calculation assumes a fully turbulent boundary layer and a hydraulically smooth ice–water boundary, which we discuss based on our borehole observations in the Supplementary Note. Melt rates computed for BH1801–04 varied from 0.56 to 2.46 m a^−1^ with a mean rate of 1.42 m a^−1^. We tested the sensitivity of the calculated melt rates to temperature, salinity, and water speed using the observed ranges. The melt rate is highly dependent on the water current (1.3 m a^−1^/10 mm s^−1^ at *T* = −1.25°C and *S*_A_ = 34.45 g kg^−1^) and water temperature (0.3 m a^−1^/0.1°C at *S*_A_ = 34.45 g kg^−1^ and *U* = 20 mm s^−1^). The melt rate at BH1803 (2.46 m a^−1^) was 40% greater than the mean over the four locations (Fig. 4). This rapid melting at BH1803 was a result of the presence at the ice base of relatively warm water (−1.2°C) at a greater depth (~360 m, *T*_f_ = −2.16°C) and a relatively strong current (Fig. 3a and Table 2).

The melt rates obtained in this study were greater than those observed using borehole-derived oceanographic data at other ice shelves in East Antarctica^15,31^. Under Fimbul Ice Shelf, the melt rate is low because WW occupies the cavity for most of the year, with MWDW only occasionally entering the cavity^15^. A borehole measurement near the front of Amery Ice Shelf in Prydz Bay revealed that basal melting at the drill site is limited year-round since basal ice is protected from melting by a layer of super-cooled water^31^. Relatively rapid basal melting obtained at the ice shelf of Langhovde Glacier is due to subshelf water temperatures of ~−1.2°C, which are warmer than those observed under other ice shelves in East Antarctica. Because the ice shelf is relatively small, subshelf water exits the cavity before the heat is fully utilized for basal melting. Consequently, the cavity is filled with water above freezing, resulting in melting along the entire ice shelf (Fig. 4). In addition, a long-term water temperature measurement near the grounding line of Langhovde Glacier showed that water temperature remains above the freezing point year-round^23^, suggesting that basal melt occurs throughout the year. These results imply that the subshelf environment of Langhovde Glacier is more directly connected to surrounding ocean conditions, and thus is more vulnerable to oceanic warming than other ice shelves in the region.

Subshelf circulation observed under the Langhovde Glacier ice shelf was characterized by the outflow of a buoyant plume along the ice base and inflow of salty warm water at depth (Figs. 3 and 4). Thermal forcing (water temperature above the in situ freezing point) at depths between 350 and 450 m was 0.69–0.77°C (Fig. 3a). This value is approximately half that observed in the Bellingshausen–Amundsen Seas^6^ and ~0.3°C higher than observed in front of Totten Glacier, East Antarctica^11^ in the same depth ranges. Ocean-water properties from previous observations indicated the existence of WDW, MWDW, ASW, and WW in Lützow-Holm Bay (Fig. 5a, b). Subshelf water temperature and salinity obtained in this study are similar to those of WW (Fig. 5b). Furthermore, water properties at BH1801–03 below ~350 m lie along a WDW-WW mixing line on the Θ-*S*_A_ diagram (Fig. 5c). These observations suggest that MWDW, that originated in the deep ocean, mixes with WW over the course of being transported through a seafloor trough into the subshelf cavity of Langhovde Glacier. The Θ-*S*_A_ diagram indicates that deep water in the Lützow-Holm Bay is warmer than that under the Langhovde Glacier ice shelf. It is likely that similar or even warmer water reaches other outlet glaciers in the region (e.g., Skallen and Telen Glaciers) (Fig. 1a), supplying heat for melting. Indeed, beneath the floating tongue of Shirase Glacier, recent measurements indicate rapid melting due to inflow of WDW^29^.

**Fig. 6:**
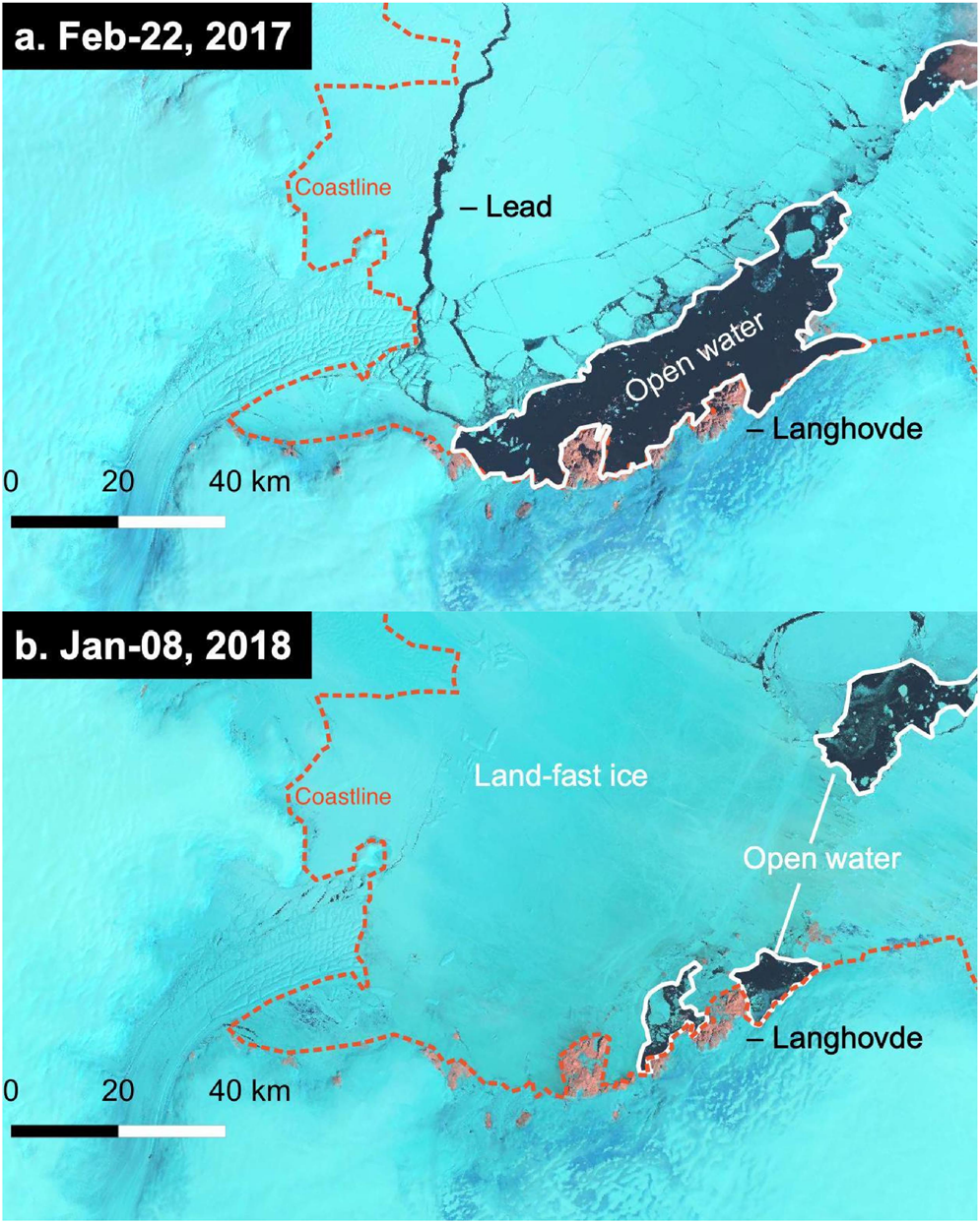
Sea–ice condition in the Lützow-Holm Bay. Landsat 8 image showing the bay on (**a**) February 22, 2017 and (**b**) January 8, 2018. White solid and orange dashed lines indicate open water areas and the coastline of glaciers and land, respectively.

We compared the water properties at BH1804 with those obtained at the same location in the previous field campaign in January 2012 (BH1201) (Fig. 1b)^23^. Interestingly, subshelf water at BH1804 in January 2018 was ~0.25°C warmer and ~0.02 g kg^−1^ saltier than the January 2012 observation (Fig. 5)^23^. We hypothesize that a change in sea ice conditions in the bay affected the subshelf conditions in 2018. Lützow-Holm Bay is usually covered by thick land-fast ice for most of the year^32^, which was the case for the period from 2006 to 2016. In summer 2016, the sea ice broke off and drifted away from the region, including the forefront of Langhovde Glacier^33^. The loss of the thick multiyear ice resulted in open water conditions near the glacier front during the following summers in 2017 and 2018 (Fig. 6). Offshore pack-ice cover was also relatively sparse during those summers. We assume that the higher temperature near the grounding line in 2018 was the result of the interaction of the open water with the atmosphere. Several studies suggest that interannual variability in the thickness of subsurface warm water is caused by wind stress^27,34^ and sea–ice production^35^. Observations near the front of Ross Ice Shelf indicated that basal melting is enhanced by inflow of warm surface water, generated in front of the ice shelf by summer insolation^16,36,37^. We are not able to specify the mechanism linking the sea ice condition and water properties in the cavity. Nevertheless, it is likely that the recent change in sea ice in Lützow-Holm Bay is at least partly affecting the ice shelf of Langhovde Glacier and other glaciers in this region.

Within the ice–ocean boundary layer at BH1801–02, water flowed toward the front, which we attribute to a buoyant plume generated by meltwater mixing with MWDW (Figs. 3 and 4). Previous observations in 2012 showed similarly stratified subshelf water near the grounding line and implied outflow along the ice base^15^. Our new data obtained in 2018 revealed the details of the stratification and flow under the entire ice shelf. A buoyant plume developed under the influence of freshwater generated by basal melting, and a buoyancy-driven current was enhanced by the relatively steep basal slope between BH1801 and 03 (Fig. 4). As a consequence of the interaction with the ice, the buoyant plume layer thickens and water flows faster as it approaches the ice shelf front (Figs. 3 and 4). This observation is consistent with a previously proposed plume theory. Basal melting near the grounding line generates a buoyant plume, which upwells alongside the ice–ocean interface toward the open ocean^38^. Such subshelf circulation is strongly affected by ocean temperature change^39^, therefore long-term monitoring is required both in the ocean and in the cavity to understand the response of the subshelf conditions to the oceanic forcing.

Near the grounding line, a complex water circulation was observed (Fig. 4). Within the same vertical distance from the ice, water near the grounding line at BH1804 was fresher and colder than that at BH1803 (Fig. 3a). As a result of the water property distributions, potential density (*σ*_Θ_) at 370-m depth increased downglacier from 27.58 kg m^−3^ at BH1804 to 27.61 kg m^−3^ at BH1803 (Fig. 3b). By assuming that the circulation in the narrow subshelf cavity near the grounding line is non-geostrophic, this horizontal density gradient has the potential to drive water flow near the grounding line, i.e., cold and freshwater flows downglacier as a buoyant plume, whereas warm and dense water fills the grounding zone. In contrast to our observations, a 10-m-thick water column in the grounding zone of Ross Ice Shelf was vertically stratified under conditions of relatively weak current (~10 mm s^−1^) and low thermal forcing (<0.1°C)^19^. The stratification was attributed to the absence of tidal mixing, and diffusive convection was suggested as a key process for driving basal melting. Thermal forcing of subshelf water at Langhovde Glacier was an order of magnitude higher than that under Ross Ice Shelf. Presumably, more rapid melting and a steeper basal ice slope generates an upslope-flowing plume, resulting in shear instabilities in the water column in the grounding zone of Langhovde Glacier. We conclude that the mixing conditions near the grounding line are controlled by the strength of basal melting, basal ice topography, and the magnitude of the tidal current, all of which will differ for different ice shelves.

The thermohaline structure and circulation presented in this study are consistent with those proposed by observation and theoretical analysis of Antarctic ice shelves^7,40^. Observations that cover the entire field of a cavity are sparse^7^, thus our data serve as an important validation of theories and models used for estimation of basal melting. Our results indicate warm water inflow into the cavity and relatively high melt rates at the base of the Langhovde Glacier ice shelf. Similar processes are expected at other Antarctic outlet glaciers in similar settings. Despite the abundance of small ice shelves distributed around Antarctica, their size means that they are not resolved in numerical models used for predicting future sea-level change^40^. Further investigation and long-term monitoring of such ice shelves is crucial for an understanding of the response of the Antarctic Ice Sheet to a changing climate.

## Methods

### Hot-water drilling and ice geometry measurements

In December 2017 and January 2018, we drilled four boreholes along a flowline of the ice shelf of Langhovde Glacier. Boreholes were drilled 0.55, 1, 1.6, and 2.5 km from the ice shelf front and referred to as BH1801, BH1802, BH1803, and BH1804 (Fig. 1). The drilling was performed at a mean rate of 42 m h^−1^ with a hot-water drilling system, which had been previously used on Langhovde Glacier^23^. The system consists of three high-pressure, hot-water machines (Kärcher HDS1000BE), a 1/2-inch diameter hose, and a winch system^41^. Meltwater was supplied to the system from a supraglacial stream or pond. Ice thicknesses and ocean bed elevation were determined to an accuracy of ±1 m, using water temperature, salinity, and pressure profiles described in the next section. At each borehole site, ice-surface elevation was measured using a dual-frequency GNSS (Leica Geosystems System 1200) with the static method using data from a reference station (GNSS Technologies, GEM-1) located on the western flank of the glacier. In order to observe lower surface ice topography, an upward-looking video camera (GoPro, HERO5) enclosed in a pressure vessel was lowered into the borehole at BH1802. Roughness of the ice surface was measured by visual inspection of the imagery.

### Oceanographic measurements and data processing

We used a CTD (conductivity, temperature, and depth) profiler (IDRONAUT Ocean Seven 304, Supplementary Fig. 1) to measure water temperature, conductivity, and depth to accuracies of 0.005°C, 7 × 10^−3^ mS cm^−1^, and 0.5 m, respectively. The conductivity was used to derive salinity to an accuracy of ~5 × 10^−3^ psu (ref. ^42^). The sample rate was 1 Hz. During the subshelf measurements the profiler, suspended on a Kevlar rope, was pulled upwards at a rate of 1 m s^−1^. To achieve a higher vertical resolution near the ice–ocean boundary, the profiler was moved more slowly, at a rate of 0.1 m s^−1^. Water depth was derived from pressure and water density computed from salinity and temperature. The magnitude and direction of the water current were measured with a current meter (JFE Advantec, AEM-USB; accuracy 10 mm s^−1^ and 2°) combined with the CTD profiler (Supplementary Fig. 1). We recorded data for 1 min with 1-s sampling intervals every 10 m in boreholes BH1801–02 and every 5 m at BH1803–04. Mean current and direction were calculated by averaging the 1-min data.

The CTD and current meter measurements were performed a day after drilling to avoid any influence from the drilling. The measurements were repeated two or three times in each borehole during periods of high and low tides to reduce the influence of tidal currents, with the exception of BH1803. As a result of a severe storm, two measurements at BH1803 took place several hours after high tide (Supplementary Fig. 2). Because of the timing of the measurements, the observed speed may be weaker than its long-term mean. However, we speculate that the magnitude of tidal currents is small because of the limited volume of the subshelf cavity particularly near the grounding line.

Water temperature and salinity were converted into conservative temperature (Θ) and absolute salinity (*S*_A_) using the standard equations^43^. To investigate mixing of meltwater and MWDW, we used a Θ-*S*_A_ diagram. As MWDW mixes with meltwater, temperature and salinity show variations on the diagram along the Gade line or meltwater-mixing line^44^, which connects water properties of MWDW and meltwater. The meltwater-mixing line was obtained using equations defined ratio of *S*_A_ to Θ changes when ice melts into seawater^44,45^, using the Gibbs SeaWater Oceanographic Toolbox of TEOS-10^43^. MWDW is a mixture of WDW and WW, thus MWDW is identified on the Θ-*S*_A_ diagram along a line connecting properties of WDW and WW. To find the lower boundary of the ice–ocean boundary layer, we analyzed the Brunt-Väisälä frequency (*N*)

2 $${N}^{2}=\frac{g}{\rho }\frac{{\rm{d}}\rho }{{\rm{d}}z},$$

where *ρ* is water density, *g* is the gravitational acceleration, and *z* is the depth below the ocean surface. Negative or zero *N*^2^ values indicate that the water column is unstable, and the stability increases as *N*^2^ takes on a larger value. In order to minimize the influence of small-scale instabilities, water density was smoothed using a Gaussian filter with a half-width of 50-m depth. A half-width of 20 m was used at BH1804 because the water column was thin. The local maximum in the calculated *N*^2^ was used to define the lower limit of the boundary layer at BH1801–03. At BH1804, we assumed the entire water column to be a boundary layer.

In addition to the data obtained in this study, we used temperature and salinity profiles available for Lützow-Holm Bay and the nearby open ocean (Fig. 5a). These measurements were obtained by the Japanese Antarctic Research Expedition between 1957 and 2018.

### Basal-melt rate

Current, temperature, and salinity measurements were used to calculate the basal-melt rate, $\dot{m}$, by solving the balance equation of heat and salt at the ice–water boundary^13,46^.

3 $${\rho }_{{\mathrm{fw}}}\dot{m}{L}_{\mathrm{i}}-{K}_{\mathrm{i}}{\left(\frac{\partial T}{\partial z}\right)}_{\mathrm{b}}=\rho {c}_{\mathrm{w}}{\gamma }_{\mathrm{T}}(T-{T}_{{\rm{0}}})$$

4 $${\rho }_{\mathrm{fw}}\dot{m}{S}_{{\rm{0}}}=\rho {c}_{\mathrm{w}}{\gamma }_{\mathrm{S}}({S}_{\mathrm{A}}-{S}_{{\rm{0}}})$$

where *T*, *S*_A_, *ρ*, and *c*_w_ are the temperature, absolute salinity, density, and specific heat capacity of water in the boundary layer, *L*_i_ and *K*_i_ are the latent heat of fusion and thermal conductivity of ice, and *ρ*_fw_ is the density of freshwater. The temperature gradient at the base of the ice (∂*T*/∂*z*)_b_ was estimated to be 0.015°C m^−1^ from borehole temperature data obtained in a previous campaign^23^. The freezing point at the ice–ocean interface *T*_0_ was given by absolute salinity at the interface *S*_0_ and pressure *P* as defined by the “gsw_t_freezing()” function^43^.

The heat and salt transfer coefficients, *γ*_T_ and *γ*_S_, for a fully developed turbulent flow over a hydraulically smooth boundary are given by

5 $${\gamma }_{\mathrm{T}}=\frac{{K}^{1/2}U}{2.12{\rm{ln}}({K}^{1/2}Re)+12.5P{r}^{2/3}}$$

and

6 $${\gamma }_{\mathrm{S}}=\frac{{K}^{1/2}U}{2.12{\rm{ln}}({K}^{1/2}Re)+12.5S{c}^{2/3}},$$

where *K* is the ice-surface drag coefficient, *Pr* and *Sc* are the Prandtl and Schmidt numbers of seawater, and *Re* is the Reynolds number defined by *Re* = *UD*/*v*, with *v* being the kinematic viscosity^13^. Measured *T*, *S*_A_, *P*, and *U* were averaged over the ice–ocean boundary layer *D* (Table 2) to numerically solve the equations (Eqs. (3) and (4)) with the function of *T*_0_ for $\dot{m}$, *T*_0_ and *S*_0_ by an iterative method. All of the parameter values used in the calculation were obtained from a previous study^30^ (Supplementary Table 1).

## Supplementary information

Supplementary info

Upward-looking movie showing the lower surface of the ice shelf at BH1802.

Description of additional supplementary files

## Data Availability

The data presented in the paper can be obtained from a data repository located at https://ads.nipr.ac.jp/dataset/A20201225-001. Temperature and salinity profiles observed in the Lützow-Holm Bay and nearby open ocean are available at https://www.jodc.go.jp, last accessed May 1, 2019. The Landsat 8 images are available at http://earthexplorer.usgs.gov, last accessed June 1, 2020.
