# Supplementary material for: Thermohaline structure and circulation beneath the Langhovde Glacier ice shelf in East Antarctica
Source: Nat Commun. 2021 Jul 9;12:4209. doi: 10.1038/s41467-021-23534-w (PMC8270922; doi:10.1038/s41467-021-23534-w)
Supplement: Supplementary file 1 — Supplementary info [file 41467_2021_23534_MOESM1_ESM.pdf]

Supplementary Information for

Thermohaline structure and circulation beneath the Langhovde Glacier ice shelf in East Antarctica

Masahiro Minowa, Shin Sugiyama, Masato Ito, Shiori Yamane, and Shigeru Aoki

Masahiro Minowa
E-mail: minowa.masahiro@gmail.com

This PDF file includes:

Supplementary Note
Supplementary Figs. 1 to 4
Caption for Supplementary Movie 1
References for SI reference citations

Other supplementary materials for this manuscript include the following:

Supplementary Movie 1

Supporting Information Text

Supplementary Note 1. Equations and parameters used for the basal melt calculation rely on two assumptions: (i) a fully turbulent boundary layer, and (ii) a hydraulically smooth ice-water boundary (1, 2). Our *in-situ* data suggest that these commonly introduced assumptions were not fully satisfied at the Langhovde Glacier borehole locations. At BH1801, 02 and 04, the water layer within 2–3 m of the ice was thermally stratified (Supplementary Fig. 4a). Water properties within this layer do not follow the meltwater-mixing line (Supplementary Fig. 4b), suggesting that heat transfer was controlled primarily by thermal diffusion as opposed to mixing due to turbulence. Thus assumption (i) was not satisfied. In contrast to these three locations, the boundary layer at BH1803 was well-mixed and satisfied the assumption (Fig. 4). A possible interpretation of the heterogeneity is local geometry of the under surface of the ice shelf. Presumably, mobility of water in the boundary layer is influenced by depressions and bumps on the ice surface formed on a variety of spatial scales. The CTD measurements at BH1803 took place several hours after high tide, thus relatively uniform water properties near the ice may be attributed to tidal current. Nonetheless, our borehole video observation at BH1802 showed ice surface dimples as evidence of melting due to turbulence (Fig. 2 and Supplementary movie).

Assumption (ii) is fulfilled when roughness of the ice surface is smaller than the laminar sublayer, which is expected to be a few millimeters thick (3, 4). The dimples observed on the ice surface were 0.05–0.1 m (Fig. 2 and Supplementary movie), suggesting the assumption is invalid. The influence of the ice surface roughness on the melt calculation is complex and beyond the reach of this study. Thus, we employed the parameters used previously and tested with observations at an Antarctic ice shelf (2). Our measurements pose a complexity of the boundary layer conditions beneath the ice shelf, but detailed discussion is difficult because of the limitation of the data in space and time. Further *in-situ* observations are required for a better understanding of physical processes at the ice-ocean boundary, which in turn contribute to an accurate basal melting estimation.

Supplementary Table 1. Summary of constants and parameters for the calculation of basal melt rate.

Parameter	Description	Value
ρ_{fw}	Density of freshwater (kg m^{-3})	1,000
ρ_{w}	Density of seawater (kg m^{-3})	1,027.5
c_{w}	Specific heat capacity of the subshelf water (J °C^{-1} kg^{-1})	3,974
L_{i}	Latent heat of fusion (J kg^{-1})	334,000
K_{i}	Thermal conductivity of ice (W °C^{-1} m^{-1})	2.1
$(\partial T/\partial z)_{\mathrm{b}}$	Ice temperature gradient at base of the ice (°C m^{-1})	0.015
γ_{T}	Heat transfer coefficient	Eq. (5)
γ_{S}	Salt transfer coefficient	Eq. (6)
Pr	Prandtl number of seawater	13.8
Sc	Schmidt number of seawater	2,432
Re	Reynolds number	UD/v
v	Kinematic viscosity of sea water (m^2 s^{-1})	1.95 $\times 10^{-6}$
K	Ice-shelf drag coefficient	2.5 $\times 10^{-3}$

Supplementary Movie 1. Upward-looking movie showing the lower surface of the ice shelf at BH1802.

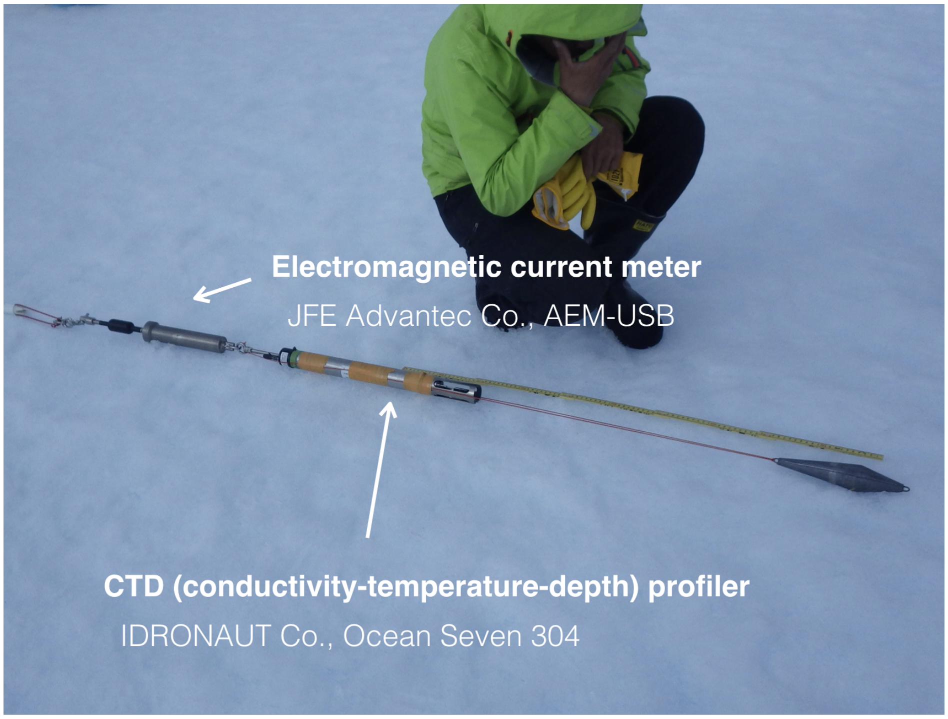

Supplementary Fig. 1. The CTD profiler and current meter used in this study.

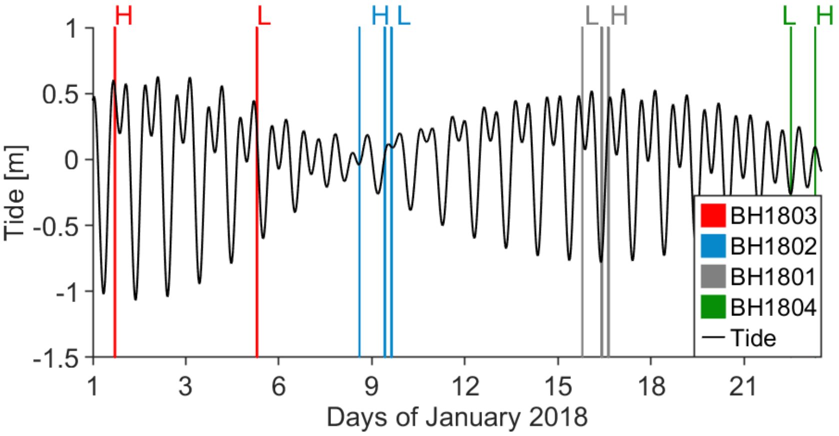

Supplementary Fig. 2. Tide and time of the oceanographic measurements. The colored vertical lines indicate the timing when we lowered the instruments twice or three times at each borehole site. The measurement dates and periods are summarized in Table 1. The measurements at around high tide (denoted by H) and low tide (denoted by L) were separately plotted in Supplementary Fig. 3. Tidal height was estimated at the glacier location using BAYTAP-G software with a tidal record at Syowa Station (5).

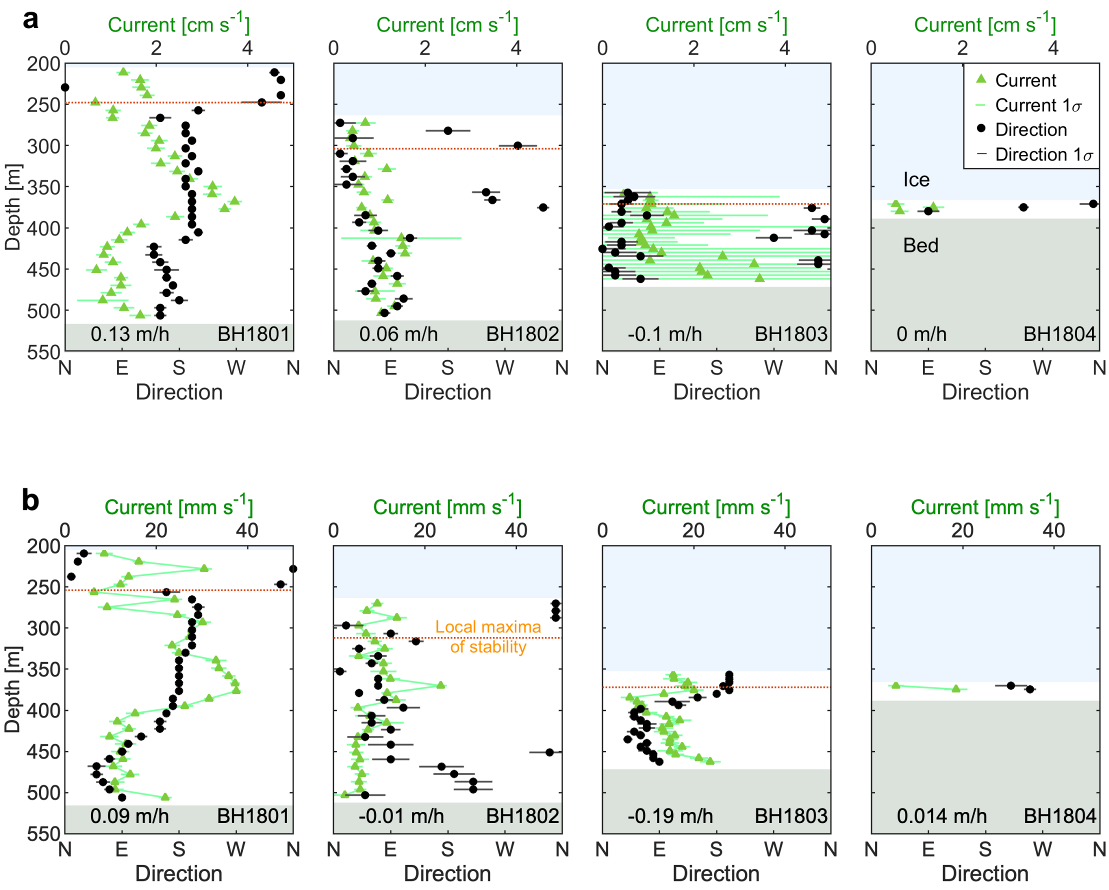

Supplementary Fig. 3. Water current (green triangles) and current direction (black dots) measured in the subshelf cavity of Langhovde Glacier January 2018. Measurements were performed twice at each borehole site approximately at **(a)** high tide and **(b)** low tide. Error bars indicate the standard deviation of the data obtained by measurements for 1 min at each depth. Horizontal orange dotted lines indicate local maxima of stability as determined in Figure 3b. Mean tidal velocities during the measurements are indicated by numbers in black.

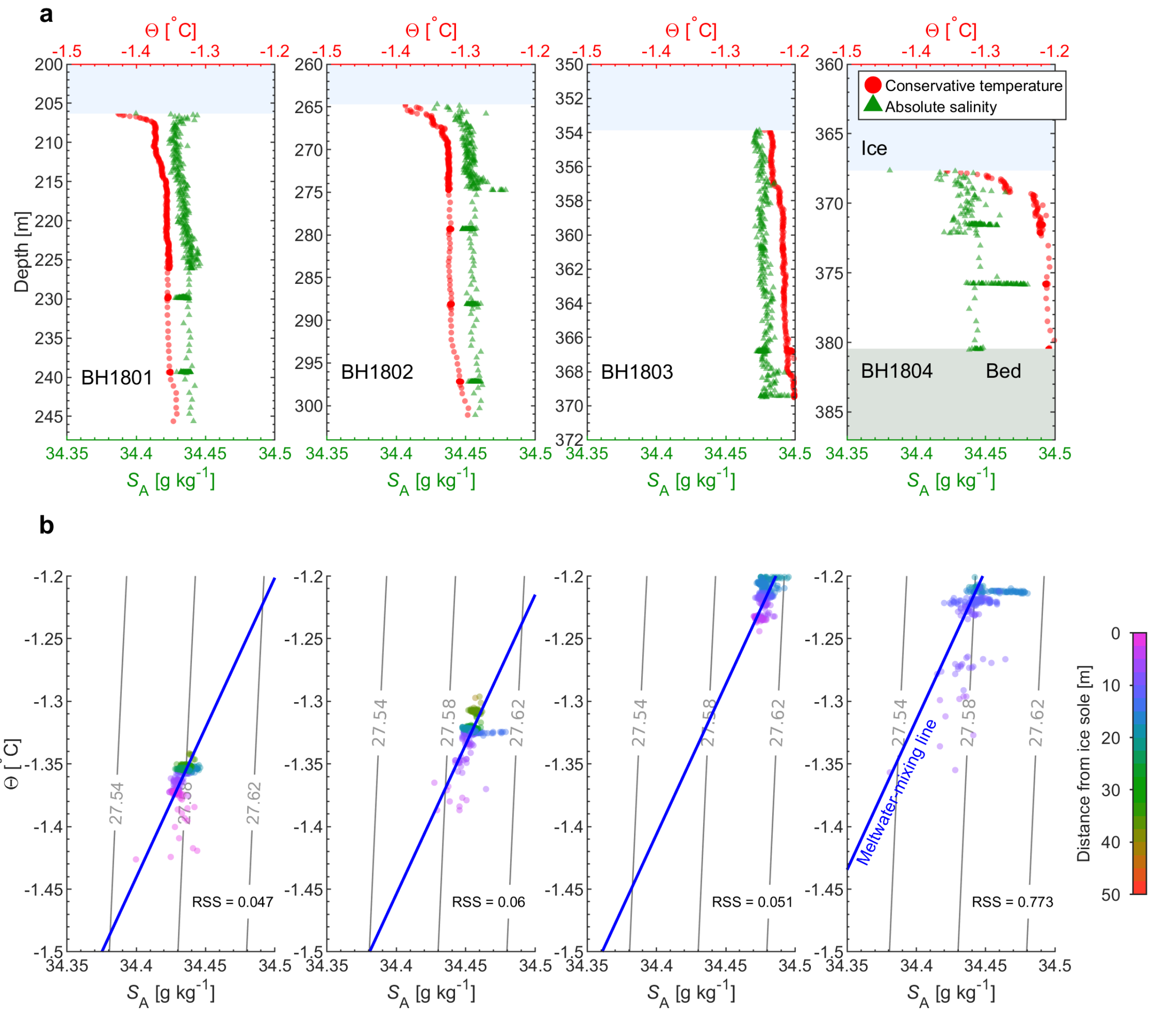

Supplementary Fig. 4. a Conservative temperature and absolute salinity variations in vicinity of the ice base. Light blue areas indicate the ice. **b** Conservative temperature–absolute salinity diagram with distance from the ice base indicated by the color scale. Blue lines are the meltwater-mixing line (gradient of 2.41 °C/(g kg^{-1})). Residual sum-of-squares (RSS) from the melt-water mixing line are shown in the panels.

Supplementary References

1. Jenkins A, Doake CS (1991) Ice-ocean interaction on Ronne Ice Shelf, Antarctica. *J. Geophys. Res.* 96(C1):791–813.
2. Jenkins A, Nicholls KW, Corr HFJ (2010) Observation and Parameterization of Ablation at the Base of Ronne Ice Shelf, Antarctica. *J. Phys. Oceanogr.* 40(10):2298–2312.
3. McPhee M (2008) *Air-ice-ocean interaction: Turbulent ocean boundary layer exchange processes*. (Springer Science & Business Media).
4. Kimura S, Nicholls KW, Venables E (2015) Estimation of ice shelf melt rate in the presence of a thermohaline staircase. *J. Phys. Oceanogr.* 45(1):133–148.
5. Minowa M, Podolskiy EA, Sugiyama S (2019) Tide-modulated ice motion and seismicity of a floating glacier tongue in East Antarctica. *Ann. Glaciol.* 60(79):57–67.
