# Supplementary material for: Thermohaline structure and circulation beneath the Langhovde Glacier ice shelf in East Antarctica
Source: Nat Commun. 2021 Jul 9;12:4209. doi: 10.1038/s41467-021-23534-w (PMC8270922; doi:10.1038/s41467-021-23534-w)
Supplement: Supplementary file 3 — Description of additional supplementary files [file 41467_2021_23534_MOESM3_ESM.docx]

Description of additional supplementary information files

Title: Supplementary Movie 1.

Description: Upward-looking movie showing the lower surface of the ice shelf at BH1802.
